# Evaluation of Plasma miR-17-5p, miR-24-3p and miRNA-223-3p Profile of Hepatitis C Virus-Infected Patients after Treatment with Direct-Acting Antivirals

**DOI:** 10.3390/jpm13081188

**Published:** 2023-07-26

**Authors:** Zehra Öksüz, Laura Gragnani, Serena Lorini, Gülhan Örekici Temel, Mehmet Sami Serin, Anna Linda Zignego

**Affiliations:** 1 Department of Pharmaceutical Microbiology, Faculty of Pharmacy, Mersin University, 33160 Mersin, Turkey; serinm@mersin.edu.tr; 2 MASVE Interdepartmental Hepatology Center, Department of Experimental and Clinical Medicine, University of Florence, Center for Research and Innovation CRIA-MASVE, AOU Careggi, 50134 Firenze, Italy; laura.gragnani@unipi.it (L.G.); serena.lorini@unifi.it (S.L.); 3 Department of Translational Research & New Technologies in Medicine and Surgery, University of Pisa, 56126 Pisa, Italy; 4 Department of Biostatistics, Faculty of Medicine, Mersin University, 33343 Mersin, Turkey; gulhan_orekici@hotmail.com

**Keywords:** hepatitis C virus, direct-acting antivirals, miR-24-3p, miR-17-5p, miR-223-3p

## Abstract

The expression of miR-223-3p, miR-17-5p, and miR-24-3p was evaluated in hepatitis C virus (HCV) patient serum samples, collected before DAA treatment and after a sustained virological response (SVR). Fifty HCV patients were stratified based on their liver damage stages into three different subgroups (21 with chronic hepatitis—CH, 15 with cirrhosis, and 14 with hepatocellular carcinoma—HCC). Considering the entire HCV population, the miRNA expression levels were significantly downregulated after the SVR compared to pre-treatment ones (*p* < 0.05). Stratifying the patients based on liver damage, the post-SVR values of the three miRNAs were significantly downregulated compared to the pre-treatment levels for both cirrhosis and HCC patients. No significant differences emerged from the analysis of the CH group. To our knowledge, this is the first study to detail the behavior of miR-223-3p, miR-17-5p, and miR-24-3p levels in patients with HCV-related CH, cirrhosis, and HCC after DAA therapy. Our findings show that HCV-infected patients have different miRNA profiles before and after treatment with DAAs, strongly suggesting that miRNAs may be involved in the pathogenesis of HCV-related damage. In this respect, the correlation observed among the three studied miRNAs could imply that they share common pathways by which they contribute the progression of HCV-induced chronic liver damage.

## 1. Introduction

The hepatitis C virus (HCV) is a major cause of serious liver disease, by which approximately 71 million people worldwide are affected [1]. In the long-term clinical history of chronic hepatitis C (CHC) infection, liver injury can range from minimal necroinflammatory changes to serious liver complications such as fibrosis, cirrhosis, and hepatocellular carcinoma (HCC) [2]. HCC is the sixth most commonly diagnosed cancer and the fourth leading cause of cancer mortality in the world [3]. In the past, the standard treatment for HCV infection was pegylated interferon (Peg-IFN)/ribavirin (RBV) until, in 2014, direct-acting antiviral (DAA) agents became available. DAA therapies, whose primary targets are non-structural proteins (HCV NS3/4A, NS5A, and NS5B), inhibit critical steps in the HCV replication cycle. The first direct-acting antiviral protease inhibitors, boceprevir and telaprevir, were approved by the United States Food and Drug Administration (FDA) in 2013. Since then, many highly effective and well-tolerated DAA regimens have been developed, which consist of various combinations of simeprevir, daclatasvir, sofosbuvir, ledipasvir/sofosbuvir, ombitasvir/paritaprevir/ritonavir plus dasabuvir, sofosbuvir/velpatasvir, elbasvir/grazoprevir, sofosbuvir/velpatasvir/voxilaprevir, and glecaprevir/pibrentasvir [4]. DAA therapy has revolutionized hepatitis C treatment by increasing sustained viral response rates (SVR) by over 90–100% [5,6]. These treatments have very low toxicity and can be administrated for short periods (two or three months); they are usually pan-genomic drugs and, because of their excellent tolerance compared to IFN therapy, assure a high level of compliance [7].

In HCV infection, host factors, as well as viral factors, play a role in the induction and maintenance of inflammation, liver fibrosis, and/or tissue regeneration. Treatment should both ensure virus clearance and improve host-related factors [8]. However, the regulation of inflammatory and fibrosis biomarkers associated with HCV eradication after DAA treatment is still unclear, and also the clinical effects of treatment with these antivirals are not yet fully known. The emergence of conflicting data in the literature regarding the increased risk of HCC in patients following treatment with DAA agents has led to controversy [9,10,11]. In the literature, some researchers have found unexpected recurrent HCC following DAA therapy [9], while other researchers have shown no significant risk [11]. This issue has led to increased clinical interest, resulting in different studies and metanalyses that have demonstrated a decrease in the HCC onset rate in HCV patients treated with DAA [12,13].

Recent studies have shown that host-related molecular factors can play an important role in determining the course of HCV infection [14]. Together with genetic predisposition, epigenetic regulators such as microRNA (miRNA) expression can make an extremely important contribution to the physiological processes as well as to the pathogenesis of several diseases. microRNAs are a class of endogenous 19–25-nucleotide long non-coding RNA molecules regulating a wide variety of genes by blocking the translation of complementary mRNAs [15]. Therefore, microRNAs have been associated with many events in a wide range of biological pathways, such as cellular development, differentiation, proliferation, metabolism, immunity, and death [16,17]. As with other viruses, HCV interacts with cellular miRNAs that can help in the replicative process. In fact, it is known that in different stages of the life cycle of HCV, some microRNAs, such as miR-122, can increase the virus replication rate and contribute to the spread of the infection in the hepatocytes [16,18]. On this basis, miR-122 has been identified as an essential host factor for new viral particle production and for the efficient replication of HCV [19], providing one of the first pieces of evidence that a host miRNA can contribute to an infectious process in humans [20].

miRNA expression is often modulated by up- or downregulation in HCV infection and its complications, such as fibrosis, cirrhosis, and HCC [21]. It has been shown that circulating miRNAs are candidate non-invasive biomarkers for the diagnosis of liver fibrosis, cirrhosis, and HCC [22,23]. In this context, the analysis of circulating miRNA expression, which can serves as biomarkers in HCV-related complications, may be important to support the long-term monitoring and clinical management of patients.

In the current study, the expression profiles of miR-17-5p, miR-24-3p, and miR-223-3p from the serum of 50 HCV patients (21 chronic hepatitis (CH), 15 cirrhosis, and 14 hepatocellular carcinoma (HCC)) were evaluated both pre- (baseline) and post-treatment with DAAs. This study aimed to analyze the levels of these three circulating miRNAs in patients with CH, cirrhosis, and HCC treated with DAAs and to show their modulation after treatment.

## 2. Materials and Methods

### 2.1. Study Population

The blood samples of 50 HCV patients, 21 with CH, 15 with cirrhosis, and 14 with HCC, obtained both before and after treatment with DAAs, were retrospectively included in this study. The serum of these patients was collected before treatment (baseline) and 6 months after the end of treatment with DAAs. The clinical follow-up of the patients was performed at the Interdepartmental Hepatology Center MaSVE (Systemic Manifestations of Hepatitis Viruses), Department of Experimental and Clinical Medicine, University of Florence. In this study, elevated liver enzymes (ALT, alanine aminotransferase; AST, aspartate aminotransferase) and the presence of HCV-RNA in serum were considered as inclusion criteria; co-infection with other viruses (such as hepatitis B virus (HBV), human immunodeficiency virus (HIV)) and the presence of other liver diseases and various comorbidities (e.g., rheumatoid arthritis, systemic lupus erythematosus, and myotonic dystrophy type 1) were exclusion criteria. The HCV RNA titer and genotype were determined as routine tests.

Liver disease was evaluated by non-invasive methods including liver elastography using FibroScan (Echos-ens, Paris, France), imaging, clinical presentation, and laboratory data; the METAVIR score system was used to assess the severity of liver disease.

The clinical and demographic data obtained from the routine examinations of the patients and the used DAAs are summarized in Table 1. The whole study was conducted according to the Declaration of Helsinki and approved by the Ethical Committee (BIO.16.014).

### 2.2. RNA Isolation and Reverse Transcription

According to the manufacturer’s protocol, RNA isolation from plasma samples was performed using the miRNeasy Serum/Plasma Advanced Isolation Kit (Qiagen, Hilden, Germany). RNA was stored at −20 °C until reverse transcription. Isolated RNA (160 nanograms for each sample) was reverse-transcribed using the M-MVL reverse transcriptase (Promega, Milan, Italy) kit. Reverse transcription was carried out using a Rotor-Gene Q Real-Time PCR System (Qiagen, Hilden, Germany) for 10 min at 25 °C, 50 min at 37 °C, and 15 min at 70 °C conditions.

### 2.3. Quantitative Real-Time PCR (RT-qPCR)

qPCR reaction was carried out in a Rotor-Gene Q Real-Time PCR System (Qiagen, Hilden, Germany) using the GoTaq^®^ Probe qPCR Master Mix (Promega, Italy) and specific TaqMan MicroRNA Assays (Applied Biosystems, Foster City, CA, USA), according to the manufacturer’s instructions. Relative expression levels of the different miRNAs were evaluated using RNU48 small-nucleolar RNA as a housekeeping RNA. Reactions were run as duplicates for each sample.

### 2.4. Statistical Analysis

Through RT-PCR, we analyzed the expression levels of miR-223-3p, miR-17-5p, and miR-24-3p. RNU48 was used as an endogenous control. Threshold cycle (Ct) data from the RT-PCR quantification cycle were analyzed via relative quantification by the 2-ΔΔCT method [24]. As descriptive statistics, numbers and percentages were given for categorical variables. The mean standard deviation values were given for the continuous variables. A paired-sample *t*-test was preferred for continuous variables and the McNemar test was preferred for categorical variables, to control for differences between pre- and post-treatment measurements in each group. The correlation coefficient was used to test the relationships between miRNA measurements, and a scatter diagram was preferred to present the visual relationships between the variables. A graph of fold changes was plotted to represent the variation between miRNAs pre-treatment versus post-treatment. *p* < 0.05 was considered statistically significant.

## 3. Results

### 3.1. Patient Characteristics

The patient population included 50 HCV patients (mean age, 67.46 ± 11.75), and 19/50 (38%) were females. Of these patients, 21 had CH (mean age, 65.90 ± 11.61), 15 had cirrhosis (mean age 66.67 ± 12.50), and 14 had HCC (mean age 70.64 ± 11.33). The most common HCV genotype was 1b (56%). Patients included in the study underwent anti-HCV IFN-free treatment with DAAs according to their genotypes (Table 1). In addition, 17 (34%) patients were treatment-naïve, whereas seven (14%) had been previously treated with Peg-IFN + RBV and 10 (20%) with Peg-IFN + RBV +/− BOC or +/− TEL. The pre-DAA treatment regimens of 16 patients (32%) were unknown. The pre- and post-treatment ALT, AST, and HCV RNA levels of the 50 HCV patients, and the different subgroups (CH, cirrhosis, and HCC), were compared and, as expected due to the different stages of liver damage, the variation was statistically significant (*p* < 0.05) (Table 1). A sustained virological response (SVR), defined as the absence of HCV RNA in the serum 12 weeks after the end of treatment, was observed in all patients after antiviral treatment with DAAs.

### 3.2. Analysis of miRNA Expression

**Figure 1:**
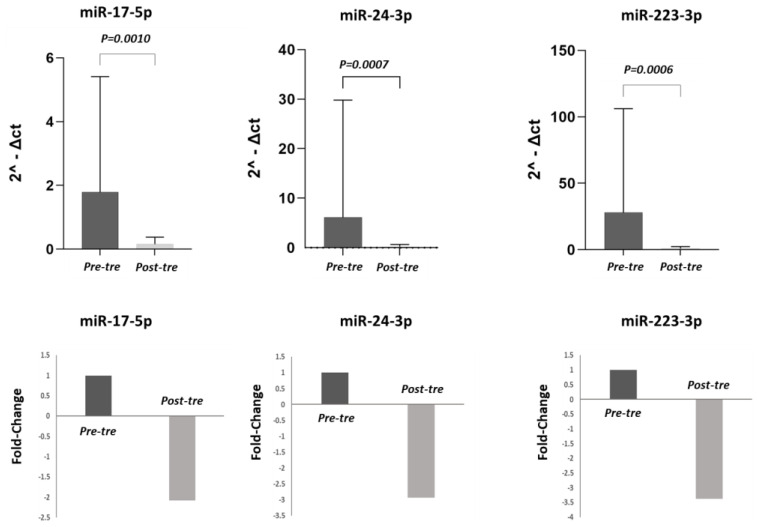
miRNA expression levels and relative fold changes in the total population of 50 HCV patients. In the first row, histograms show the miRNAs levels in the total population of HCV patients pre- and post-treatment. The miRNA levels were calculated in terms of relative expression using RNU48 as a housekeeping gene. In the second row, the fold changes are shown for each miRNA, comparing pre- and post-treatment values. Considering the pre-treatment value as a calibrator, after the therapy, the expression levels of miR-17-5p, miR-24-3p, and miR-223-3p were downregulated 2.08-fold, 2.94-fold, and 3.38-fold, respectively. (Pre-tre: pre-treatment, Post-tre: post-treatment, 2^ΔCt: 2 elevated at delta Ct).

**Figure 2:**
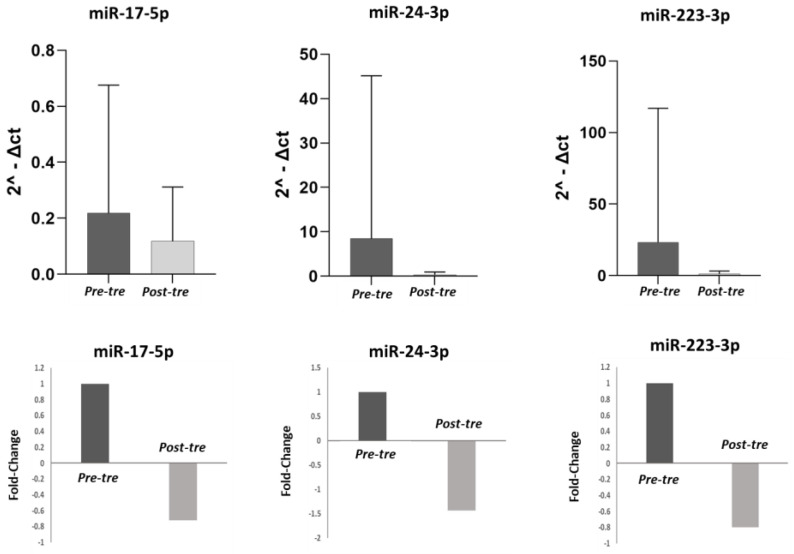
miRNA expression levels and relative fold changes in the group of patients with HCV-related chronic hepatitis. In the first row, histograms show the miRNA levels in the chronic hepatitis (CH) group (21 patients) pre- and post-treatment. The miRNA levels were calculated in terms of relative expression using RNU48 as a housekeeping gene. In the second row, the fold changes are shown for each miRNA, comparing pre- and post-treatment values. Considering the pre-treatment value as a calibrator, after the therapy, the expression levels of miR-17-5p, miR-24-3p, and miR-223-3p were downregulated 0.72-fold, 1.43-fold, and 0.80-fold, respectively. (Pre-tre: pre-treatment, Post-tre: post-treatment, 2^ΔCt: 2 elevated at delta Ct).

**Figure 3:**
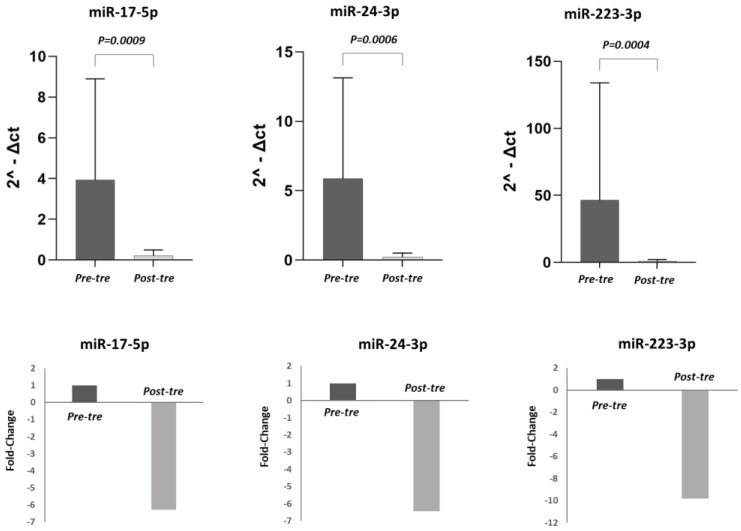
miRNA expression levels and relative fold changes in the group of patients with HCV-related cirrhosis. In the first row, histograms show the miRNA levels in the cirrhosis group (15 patients) pre- and post-treatment. The miRNA levels were calculated in terms of relative expression using RNU48 as a housekeeping gene. In the second row, the fold changes are shown for each miRNA, comparing pre- and post-treatment values. Considering the pre-treatment value as a calibrator, after the therapy, the expression levels of miR-17-5p, miR-24-3p, and miR-223-3p were downregulated 6.27-fold, 6.45-fold, and 9.84-fold, respectively. (Pre-tre: pre-treatment, Post-tre: post-treatment, 2^ΔCt: 2 elevated at delta Ct).

**Figure 4:**
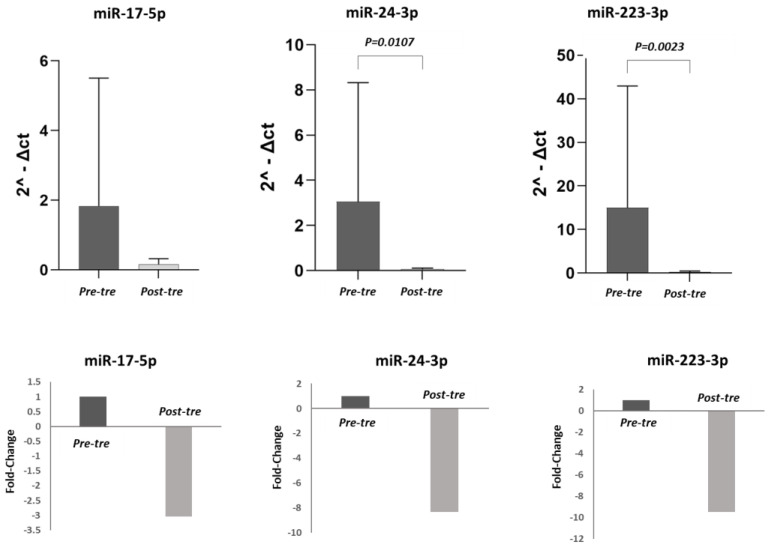
miRNA expression levels and relative fold changes in the group of patients with HCV–hepatocellular carcinoma. In the first row, histograms show the miRNA levels in the hepatocellular carcinoma (HCC) group (14 patients) pre- and post-treatment. The miRNA levels were calculated in terms of relative expression using RNU48 as a housekeeping gene. In the second row, the fold changes are shown for each miRNA, comparing pre- and post-treatment values. Considering the pre-treatment value as a calibrator, after the therapy, the expression levels of miR-17-5p, miR-24-3p, and miR-223-3p were downregulated 3.05-fold, 8.33-fold, and 9.51-fold, respectively. (Pre-tre: pre-treatment, Post-tre: post-treatment, 2^ΔCt: 2 elevated at delta Ct).

In this study, the expression levels of pre-treatment miR-223-3p, miR-17-5p, and miR-24-3p were compared with post-treatment levels in both the total population of 50 HCV patients and in the different patient subgroups (CH, cirrhosis, HCC). In the 50 HCV patients, the expression levels of miR-17-5p, miR-223-3p, and miR-24-3p were significantly lower post-treatment compared to pre-treatment (*p* < 0.05). (Figure 1). Considering the different groups, in the CH patients (n = 21), the decrease in the expression of these three miRNAs post-treatment did not reach a significant difference (*p* > 0.05) (Figure 2). By contrast, in the cirrhosis patient group (n = 15), the expression levels of miR-17-5p, miR-24-3p, and miR-223-3p were significantly higher pre-treatment compared to post-treatment (*p* < 0.05) (Figure 3). In the HCC patient group (n = 14), miR-24-3p and miR-223-3p were significantly downregulated post-treatment compared to pre-treatment; concerning miR-17-5p expression, the level decreased without reaching statistical significance, (*p* > 0.05) (Figure 4). The expression levels and *p* values of the studied miRNAs are shown in Figure 1, Figure 2, Figure 3 and Figure 4 together with the fold change analysis obtained by considering the pre-treatment value as a calibrator.

### 3.3. Pre- and Post-Treatment miRNA Expression Correlation

**Figure 5:**
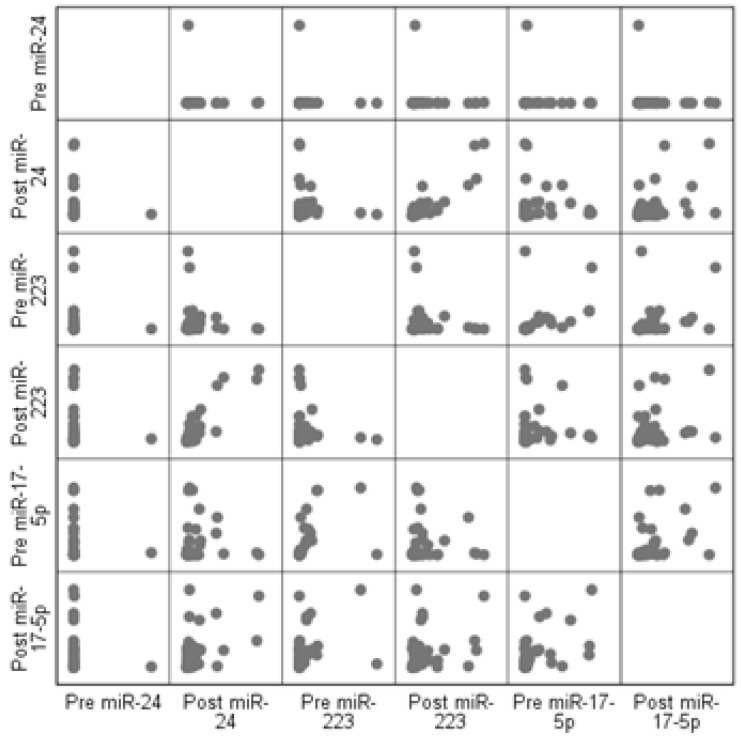
Pre- and post-treatment miRNA scatter diagrams. Symmetrical view of miR-17-5p, miR-24-3p and miR-223-3p expression diagram pre and post-treatment in a total of 50 HCV patient groups.

The correlation of miR-17-5p, miR-24-3p, and miR-223-3p expression pre- and post-treatment was examined in the total group of 50 HCV patients. A strong linear correlation was found between the value of pre-treatment miR-24-3p and both pre-treatment miR-223-3p (*p*< 0.001) and pre-treatment miR-17-5p (*p* < 0.001). In addition, a high level of linear correlation was found between the values of post-treatment miR-24-3p and post-treatment miR-223-3p (*p* < 0.001). A strong linear correlation was found between the values of post-treatment miR-223-3p and post-treatment miR-17-5p (*p* = 0.001). Similarly, a strong linear correlation was found between the pre-treatment miR-17-5p and the pre-treatment miR-223-3p values (*p*< 0.001). However, a moderate linear relationship was detected between pre-treatment miR-24-3p and post-treatment miR-17-5p (*p* = 0.001), post-treatment miR-24-3p and post-treatment miR-17-5p (*p =* 0.002), pre-treatment miR-223-3p and post-treatment miR-17-5p (*p* = 0.003), and pre-treatment miR-24-3p and post-treatment miR-17-5p (*p* < 0.001). Pre- and post-treatment and miRNA expression correlation comparisons are shown in Table 2 and Figure 5.

## 4. Discussion

The risk of developing liver-related complications in HCV-infected patients who achieve an SVR after treatment with DAAs is controversial [25]. Therefore, patients with HCV, particularly those with advanced liver damage, should still be monitored for signs of liver disease progression, even if they achieve an SVR after DAA therapy [26]. Recently, it has been shown that miRNAs may play a role in the regeneration or progression of liver damage in patients with chronic HCV infection [27,28]. In this study, we investigated the expression of miR-17-5p, miR-24-3p, and miR-223-3p in the sera of HCV-infected patients after treatment with DAAs to determine the effect of DAA treatment and viral eradication on host factors reputed to play an important role in the pathogenesis of liver damage. To perform this analysis, circulating levels of plasma miR-17-5p, miR-24-3p, and miR-223-3p were measured in matched samples of CH, cirrhosis, and HCC patients pre- (baseline) and post-antiviral therapy (6 months after treatment). Our findings showed that the expression of the miRNAs included in the study was downregulated after DAA treatment in the total population of HCV patients, with different patterns of expression in different HCV-related conditions: CH, cirrhosis, and HCC.

In fact, the miR-17-5p expression level was found to be significantly downregulated in the post-treatment period compared to pre-treatment with DAAs in the cirrhosis group, but not in the HCC one. miR-17-5p, a member of the miR-17-92 polycistron family, is overexpressed in many cancers, including HCC [22,29,30]. A study revealed that miR-17-5p has an important role in the carcinogenesis and development of HCC by significantly activating the p38 mitogen-activated protein kinase (MAPK) pathway and increasing the phosphorylation of heat shock protein 27 (HSP27) [31]. Chen et al. reported that miR-17-5p was upregulated in patients with HCC and may be a prognostic biomarker for HCC [32]. In a study by Yu et al., they showed that the serum miR-17-5p levels of patients with cirrhosis were significantly higher than those of healthy controls, and that the inhibition of this miRNA led to the suppression of hepatic stellate cell proliferation induced by transforming growth factor-β1 (TGF-β1) [33]. In addition, in a previous study that we performed, in which we detected miRNAs with significant expression at all stages of the disease, from CHC to cirrhosis and HCC, miR-17-5p levels were upregulated in both HCV-related cirrhosis and HCV-related HCC patients compared to healthy controls [22]. In a recent study that we conducted in the Turkish population, we examined the serum levels of this miRNA in CH patients who achieved an SVR after treatment with ombitasvir/paritaprevir/ritonavir + dasabuvir ± ribavirin [34]. Serum miR-17-5p levels were found to be significantly downregulated post-treatment compared to pre-treatment, thus supporting the present study conducted in the Italian population.

In recent studies, the role of miR-24-3p, which showed differences in expression in the initiation, progression, and metastasis of various cancer types [35], was confirmed also in HCC [36,37]. Zeng et al. [36] showed that the upregulation of miR-24-3p was inversely correlated with that of CASC2 and that it reversed growth inhibition in HCC cells. Dong et al. [37] reported that miR-24-3p played an important role in the initiation and progression of HCC by targeting metallothionein 1M. In a previous study by our group, the expression of this miRNA was shown to be upregulated in HCV-associated HCC patients compared to a healthy control group, in accordance with the literature [22]. In addition, in the study that we conducted in the Turkish population, it was found that the expression of miR-24-3p decreased in HCV patients who achieved an SVR after treatment with ombitasvir/paritaprevir/ritonavir + dasabuvir ± ribavirin [34]. Consistent with our previous results, in the present study performed in the Italian population, miR-24-3p expression was significantly downregulated after treatment with DAAs in the total population of HCV patients, as well as in the cirrhosis and HCC subgroups. After DAA treatment, the decreased expression levels of miR-17-5p and miR-24-3p (miRNAs upregulated in advanced stages of hepatitis C) in patients with cirrhosis and HCC suggest that treatment with new-generation DAAs positively affects the immunopathogenesis of these conditions. By contrast, Hyrina et al. [38] reported that miR-24 and miR-223 levels were significantly increased in patients who obtained an SVR with IFN-based antiviral treatment (Peg-IFN-α and RBV ± boceprevir (BOC) or telaprevir (TPV)), strongly suggesting the opposite effect of the pro-inflammatory IFN cytokine, possibly in combination with various factors, such as the genetic background and the different severity stages of liver disease.

Another miRNA emerging from the HCV-related HCC literature is miR-223-3p. miR-223-3p, targeting the pyrin alanine-containing NOD-like receptor family 3 (NLRP3) in HCC cells, has been shown to play an important role in this tumor’s biology, and it may be useful to ascertain the prognosis of the liver damage related to HCV infection [39]. Shaker et al. [40] showed that miR-223 was upregulated in advanced fibrosis (≥F3) compared to HCV-related early stages of fibrosis in Egyptian patients. Moreover, another study reported that this miRNA was upregulated in HCV-related HCC patients compared to healthy controls [41]. In contrast, our study group [22] and several others [42,43] previously reported that miR-223-3p is downregulated in HCC and may be a potential biomarker for the diagnosis of this type of cancer. On the other hand, a recent study highlighted that the expression of circulating miRNA-223 showed no significant difference between HCV, HCV-related cirrhosis, and HCC [44]. Our data showed that miR-223-3p expression, similarly to miR-24-3p, was significantly downregulated after treatment with DAAs in patients affected by cirrhosis and in subjects with HCC, but not in patients with CH. Interestingly, in our previous study performed in a Turkish population of HCV patients, it was shown that the expression of this miRNA in HCV patients who achieved an SVR after treatment with ombitasvir/paritaprevir/ritonavir + dasabuvir ± ribavirin was decreased, even if the difference did not reach statistical significance [32]. This may be due to the difference in the number of patients included in the study, to the geographical origin of the populations that we analyzed (Turkish and Italian), to the HCV genotypes affecting the patients included in the study, and to the different methods used to process the ex vivo samples and for the miRNA extraction, detection, and normalization.

Another striking result regarding the miRNAs included in the study was the high level of correlation observed in the plasma levels of circulating miR-17-5p, miR-24-3p, and miR-223-3p pre-treatment with DAAs. This suggests that there may be a close link between these miRNAs, which may result from common pathways or target genes, or that they contribute to shared mechanisms.

## 5. Conclusions

To our knowledge, this is the first study detailing the potential diagnostic value of miR-17-5p, miR-24-3p, and miR-223-3p in patients with chronic HCV-related liver diseases (CH, cirrhosis, and HCC) after DAA therapy. In conclusion, our findings show that HCV-infected patients have different microRNA profiles before and after treatment with DAAs and that the differences in their deregulation seem to be associated with particular steps in the progression of liver damage. In particular, this study suggests that miR-17-5p and miR-24-3p play an important role in the pathogenesis of HCV-related CH, cirrhosis, and HCC and may be potential therapeutic targets. In other words, this study provides preliminary data to elucidate the pathogenetic pathways underlying the progression of HCV-related liver damage, suggesting the importance of further studies clarifying, at the molecular level, the roles and mechanisms of these miRNAs in HCV infection. This will be made possible by analyzing larger populations of patients and taking into account the different grades of progression (both hepatic and extrahepatic) of the HCV chronic infection.

## Figures and Tables

**Table 1:** Demographic and clinical information of patients.

	Total HCV Population (*n* = 50)	CH Patients (*n* = 21)	Cirrhotic Patients (*n* = 15)	HCC Patients (*n* = 14)	*p*
Age (mean ± SD)	67.46 ± 11.75	65.90 ± 11.61	66 ± 12.61	70.64 ± 10.91	
Gender					
Male	31 (62)	6 (28)	6 (40)	7 (50)	
Female	19 (38)	15 (72)	9 (60)	7 (50)	
Genotype, n (%)					
1a–1b	5 (10)–28 (56)	3 (14)–7 (33)	1 (6)–9 (60)	1 (7)–12 (85)	
2–2a/2c	9 (18)–4 (8)	7 (33)–3 (14)	2 (13)–1 (6)	-	
3a–3c	3 (6)–1 (2)	1 (4)–0	1 (6)–1 (6)	1 (7)–0	
DAA treatment (%)					
Harvoni	19 (38)	7 (33)	5 (33)	7 (50)	
SOF + RBV	15 (30)	10 (47)	4 (26)	1 (7)	
SOF + RBV + DAC	7 (14)	-	4 (26)	3 (21)	
DAS + OMB + ABT 450	4 (8)	-	2 (13)	2 (14)	
Maviret	4 (8)	4 (19)	-	-	
Zepatier	1 (2)	-	-	1 (7)	
ALT (IU/L)					
Pre-treatment	90.78 ± 72.70	95.32 ± 89.67	75.67 ± 45.19	97.57 ± 68.84	<0.001 * 0.002 ** 0.001 *** 0.003 ****
Post-treatment	25.93 ± 9.88	22.60 ± 7.18	24.25 ± 6.64	31.86 ± 12.87	
AST (IU/L)					
Pre-treatment	88.47 ± 63.06	92.47 ± 84.41	81.92 ± 36.98	88.64 ± 48.71	<0.001 * 0.001 ** <0.001 *** <0.001 ****
Post-treatment	25.04 ± 10.97	22.68 ± 9.87	27.33 ± 11.78	26.29 ± 11.84	
HCV RNA (IU/mL)					
Pre-treatment	3,054,496.80 ± 4,604,619.523	3,428,032.75 ± 6,306,331.477	1,865,182.82 ± 1,847,452.949	3,455,335.00 ± 3,070,385.284	<0.001 * 0.003 ** 0 < 0.001 *** 0.001 ****
Post-treatment	0	0	0	0	

CH: chronic hepatitis C, HCC: hepatocellular carcinoma, ALT: alanine aminotransferase, AST: aspartate aminotransferase, DAS: dasabuvir, omb: ombitasvir, rbv: ribavirin, sof: sofosbuvir, dac: daclatasvir. Statistical significance is shown as * *p* < 0.05, pre- and post-treatment comparison in the total HCV population; ** *p* < 0.05, pre- and post-treatment comparison in CH patients; *** *p* < 0.05, pre- and post-treatment comparison in cirrhotic patients; **** *p* < 0.05, pre- and post-treatment comparison in HCC patients.

**Table 2:** Comparison of pre- and post-DAA treatment miRNA expression correlations.

miRNA	*p*	Pre-miR-24-3p	Post-miR-24-3p	Pre-miR-223-3p	Post-miR-223-3p	Pre-miR-17-5p	Post-miR-17-5p
Pre-miR-24-3p	*r*	1.000	0.295	0.780	0.385	0.794	0.517
	*p*	.	0.037 *	<0.001 *	0.006 *	<0.001 *	<0.001 *
Post-miR-24-3p	*r*		1.000	0.183	0.741	0.212	0.427
	*p*		.	0.204	<0.001 *	0.139	0.002 *
Pre-miR-223-3p	*r*			1.000	0.272	0.859	0.412
	*p*			.	0.056	<0.001 *	0.003 *
Post-miR-223-3p	*r*				1.000	0.227	0.460
	*p*				.	0.113	0.001 *
Pre-miR-17-5p	*r*					1.000	0.473
	*p*					.	0.001 *
Post-miR-17-5p	*r*						1.000
	*p*						.

Statistical significance is shown as * *p* < 0.05, r: Pearson correlation value.

## Data Availability

Data can be requested from the corresponding author.
